# Intra-Assessment Resting Metabolic Rate Variability Is Associated with Cardiometabolic Risk Factors in Middle-Aged Adults

**DOI:** 10.3390/jcm12237321

**Published:** 2023-11-26

**Authors:** Juan M. A. Alcantara, Francisco J. Osuna-Prieto, Manuel J. Castillo, Abel Plaza-Florido, Francisco J. Amaro-Gahete

**Affiliations:** 1 Department of Health Sciences, Institute for Innovation & Sustainable Food Chain Development, Public University of Navarre, 31006 Pamplona, Spain; 2 Navarra Institute for Health Research, IdiSNA, 31008 Pamplona, Spain; 3 Centro de Investigación Biomédica en Red Fisiopatología de la Obesidad y Nutrición (CIBERobn), Instituto de Salud Carlos III, 28029 Madrid, Spain; amarof@ugr.es; 4 Research Institute in Health Pere Virgili, University Hospital of Tarragona Joan XXIII, 43005 Tarragona, Spain; fjosunaprieto@ugr.es; 5 Department of Physical Education and Sports, Faculty of Sports Science, Sport and Health University Research Institute (iMUDS), University of Granada, 18071 Granada, Spain; 6 EFFECTS-262 Research Group, Department of Physiology, Faculty of Medicine, University of Granada, 18071 Granada, Spain; mcgarzon@ugr.es; 7 Instituto de Investigación Biosanitaria, IBS.GRANADA, 18014 Granada, Spain; 8 Pediatric Exercise and Genomics Research Center, Department of Pediatrics, School of Medicine, University of California at Irvine, Irvine, CA 92617, USA; aplazafl@hs.uci.edu

**Keywords:** IC, CPX Ultima CardiO2, sexual dimorphism, health status, cardiovascular diseases, cardiometabolic risk factors

## Abstract

The intra-assessment resting metabolic rate variability is related to cardiometabolic health, as suggested by previous literature. We studied whether that variability (expressed as coefficient of variation [CV; %]) for oxygen consumption (VO_2_), carbon dioxide production (VCO_2_), respiratory exchange ratio (RER), and resting energy expenditure (REE) is similar between men and women, and if is similarly associated with cardiometabolic risk factors. Gas exchange in 72 middle-aged adults was measured by indirect calorimetry. Anthropometrics and body composition, cardiorespiratory fitness, circulating cardiometabolic risk factors, and heart rhythm parameters were also determined. Men and women presented similar intra-assessment resting metabolic rate variability (all *p* > 0.05). Notably, in men, CV for RER was positively associated with BMI and adiposity (both standardized β = 0.35, Ps ≤ 0.048), while CVs for VO_2_, VCO_2_, and REE were negatively associated (standardized β ranged from −0.37 to −0.46, all *p* ≤ 0.036) with cardiometabolic risk factors. In women, CVs for VCO_2_ and REE were negatively associated with adiposity (both standardized β = −0.36, Ps ≤ 0.041) and cardiometabolic risk Z-score (standardized β = −0.40 and −0.38, respectively, Ps ≤ 0.05). In conclusion, intra-assessment resting metabolic rate variability could be considered an indicator of cardiometabolic health in middle-aged adults.

## 1. Introduction

Resting metabolic rate is of notorious relevance in metabolism studies [1]. It represents the minimal energy required to maintain homeostasis and constitutes a significant portion of total daily energy expenditure, especially in sedentary individuals [2]. Indirect calorimetry—particularly the metabolic cart system—is the gold-standard tool for assessing resting metabolic rate, and thus, resting energy expenditure (REE) [3,4]. This technique accurately measures resting gas exchange and allows the study of potential variations in REE across species, sexes, and within or between days [5,6,7]. The reproducibility of REE assessed through metabolic cart ranges between 3% and 8%, establishing its reliability [8].

In resting metabolic rate determinations, the intra-assessment variability is usually expressed using the coefficient of variation (CV) for oxygen consumption (VO_2_), carbon dioxide production (VCO_2_), respiratory exchange ratio (RER), and REE [9]. In this regard, the variability in REE within species is closely linked to body-size-related parameters, with body mass as its main determinant [5,6,7,8]. However, beyond this factor, REE variation in mammals has practical implications for behavioral traits, functional capacity, and other health-related parameters [10,11]. Recently, Halsey et al. [12] observed greater variability in energy expenditure components in men vs. women (even considering similar ages and body-size-related parameters [e.g., fat and fat-free masses]). Similarly, our recent research demonstrated a greater intra-assessment variability in VO_2_, VCO_2_, and REE in young men compared to their women counterparts [9].

The CV for VO_2_, VCO_2_, and RER has raised a growing interest in resting metabolic rate assessments since they are considered to be “gas exchange stability indicators” [13]. Indeed, these CVs are widely employed to select the resting metabolic rate data for the estimation of REE, RER, and substrate utilization [14]. Previous studies found that body mass index (BMI) influences the intra-assessment variability [15]. Furthermore, in a study conducted on ill patients, the authors noted that 55% of the sample yielded a high intra-assessment resting metabolic rate variability [16]. Based on these findings, it has been suggested that the worse the health status, the greater the intra-assessment resting metabolic rate variability [17,18,19,20,21,22]. However, this theory remains mostly unexplored in humans. To our knowledge, our recent study was the first considering the intra-assessment resting metabolic rate variability (approached as the CV for VO_2_, VCO_2_, RER, and REE) as potential non-invasive markers of cardiometabolic risk in young men but not in women [9]. Thus, we suggested the presence of sexual dimorphism in the relationship between intra-assessment resting metabolic rate variability and cardiometabolic risk factors among young adults [9]. Moreover, in the same work [9], we proposed the CV for RER as a non-invasive marker of cardiometabolic risk, as we detected that intra-assessment RER variability was associated with heart rate and heart rate variability [23,24,25,26]. Energy expenditure decreases greatly with aging and lifespan [27]. Therefore, whether the intra-assessment variability of REE could be considered a non-invasive cardiometabolic health indicator in older populations remains unknown.

The present study explores whether the intra-assessment resting metabolic rate variability is dependent on the individual’s sex, and if such variability is potentially associated with health-related markers (i.e., anthropometry and body composition, cardiorespiratory fitness, classical circulating cardiometabolic risk factors, and heart rhythm) in middle-aged adults. Based on our previous study [9], we hypothesized that in middle-aged adults, (i) men will present a higher CV for VO_2_, VCO_2_, RER, and REE compared to women; and (ii) the intra-assessment resting metabolic rate variability parameters will be associated with an adverse cardiometabolic profile.

## 2. Materials and Methods

### 2.1. Study Participants

This cross-sectional study used baseline (pre-intervention) data from the FIT-AGEING randomized controlled trial (ClinicalTrials.gov ID: NCT03334357; https://clinicaltrials.gov/study/NCT03334357 [accessed on 7 September 2023]) [28]. A total of 72 middle-aged adults were included (see Figure S1). All participants provided written informed consent prior to their enrollment (see Institutional Review Board Statement below). Briefly, the inclusion/exclusion criteria were (i) being sedentary (less than 20 min of moderate-intensity physical activity on 3 days per week); (ii) no body weight (BW) fluctuations equal to or larger than 5 kg over the last 5 months; (iii) not being enrolled in a weight loss program; (iv) not suffering from acute or chronic illness; and (v) not being a smoker.

### 2.2. Resting Gas Exchange Measurement

Gas exchange was measured for 30 min using a CPX Ultima CardiO2 metabolic cart (Medical Graphics Corp, St. Paul, MN, USA) while participants were at rest, lying in the supine position on a bed, and awake. Gas exchange was collected using a Directconnect™ low-flow sensor (Medical Graphics Corp, St. Paul, MN, USA) attached to a face mask. Before each gas exchange measurement, metabolic cart flow and gas analyzers were calibrated strictly following the manufacturer’s instructions. Assessments were conducted in the morning and after an overnight fast (12 h). In addition, the participants were instructed to not elicit moderate or vigorous physical activity before the test (24 and 48 h, respectively). In this regard, participants commuted to the laboratory by motorized vehicle or by public transportation. Before the gas exchange measurement, an acclimation period (at least 20 min) was performed, as recommended [13].

After gas exchange measurement, data were downloaded at a sampling frequency of 1 min. Then, following current recommendations [13], we excluded the data from the first 5-minute period, and the remaining data were used for calculations. We used the equation proposed by Weir [29] for estimating REE and computed the RER as the VCO_2_/VO_2_. Finally, the CVs for VO_2_, VCO_2_, RER, and REE were calculated and expressed as percentages for each subject (e.g., CV for VCO_2_: VCO_2_ standard deviation during the remaining 25 min data/VCO_2_ average during the remaining 25 min data) × 100)). Extended and graphical information are presented elsewhere [9].

### 2.3. Maximum-Effort Walking Graded Exercise Test

The cardiorespiratory fitness (i.e., peak VO_2_ uptake) was assessed by indirect calorimetry (using the same previously mentioned metabolic cart) while participants performed a maximum-effort walking graded exercise protocol [30]. The metabolic cart was calibrated before each test. Participants were instructed to avoid stimulant substance(s) before the study visit, not to eat (3–5 h), and not to perform any moderate and/or vigorous physical activity (24 and 48 h, respectively). The maximum-effort graded exercise test consisted of walking at 5.3 km/h on a treadmill (H/P/cosmos pulsar; H/P/cosmos sports and medical GmbH, Nussdorf-Traunstein, Germany) with subsequent increments of the slope (1%) every 1-minute period until volitional exhaustion.

After the maximum-effort walking graded exercise protocol, data was downloaded in a sampling frequency of 5 sec. We screened the entire set of data collected to determine the highest (i.e., peak) VO_2_ uptake value. Then, we averaged the highest VO_2_ uptake value and the immediately prior and following 5-second values (a 15-second period) to be used as the cardiorespiratory fitness. To avoid possible measurement errors that may influence the results, we screened the data set from the 2nd to the 10th subsequent largest VO_2_ uptake value. Finally, we expressed cardiorespiratory fitness as absolute peak VO_2_ uptake values (milliliters per minute [mL/min]), as the peak VO_2_ uptake-to-BW ratio (mL/kg BW/min), and as the peak VO_2_ uptake-to-lean mass ratio (mL/kg lean mass [LM]/min).

### 2.4. Blood Pressure Measurements and Circulating Cardiometabolic Risk Factors

Blood pressure (BP) was assessed twice (HEM 705 CP; Omron Healthcare Co., Kyoto, Japan), in the right arm, and its average was used for analysis.

Concerning the blood circulating cardiometabolic risk factor assessment, in fasting and resting conditions, we obtained serum by collecting blood samples (Vacutainer^®^ SST™ II Advance tubes; Becton Dickinson, Plymouth, UK). Subsequently, the tubes were centrifuged, aliquoted, and stored (−80 °C) for later analyses.

Fasting cholesterol levels, high-density lipoprotein cholesterol (HDL-C) and low-density lipoprotein cholesterol (LDL-C), triglycerides, and glucose were quantified using spectrophotometry (Beckman Coulter model AU5800; Brea, CA, USA). We assessed fasting insulin levels (chemiluminescence immunoassay, UniCel DxI 800 paramagnetic particles; Beckman Coulter, Brea, CA, USA), and subsequently calculated the homeostatic model assessment of insulin resistance index (HOMA index) as fasting glucose × fasting insulin/22.5.

We computed a cardiometabolic risk Z-score (referred to as cardiometabolic risk Z-score 1) by employing conventional metabolic syndrome indicators. The calculation procedure involved the determination of individual Z-scores for each marker using the formula (value − mean)/standard deviation. For HDL-C, the values were inverted (multiplied by −1), resulting in higher Z-score values indicating greater risk. Cardiometabolic risk Z-score 1 was integrated as the sum of individual Z-scores for glucose, triglycerides, HDL-C, BP, and waist circumference divided by 5. Then, we also created a second score (referred to as cardiometabolic risk Z-score 2), adding the cholesterol, the LDL-C, the insulin, and the HOMA index to the parameters included in Z-score 1.

### 2.5. Resting Heart Rhythm Assessments

Heart rhythm data were captured over a 15-minute interval using a Polar RS800CX (Polar Electro, Kempele, Finland), with participants lying supine on a bed. This assessment preceded the gas exchange measurement, which occurred in the same room. Participants were instructed to remain awake, motionless, and silent during the recording.

Data from the heart rhythm were processed using the free version of the Kubios software (v.3.0.0, HRV analysis, University of Eastern, Finland). The initial 5 min of data were excluded and a subsequent 5-minute period was manually chosen as detailed elsewhere [31]. Within this 5-minute window, Kubios’ medium threshold-based artifact correction level was applied [31]. R-R interval series were sampled at 1000 Hertz, detrended using the prior smoothness method (alpha = 500 ms) and a cubic interpolation (4 Hertz rate).

The heart rhythm data were used to derive the resting mean heart rate (HR). Subsequently, four vagal-related heart rate variability (HRV) variables were extracted from both time and frequency domains [23]. The squared root of the mean of the sum of the squares of successive R-R interval differences (RMSSD), the standard deviation of all normal R-R intervals (SDNN), the percentage of pairs of adjacent R-R intervals differing by more than 50 milliseconds (pNN50), and the power of the high-frequency band (HF; 0.15–0.4 Hertz) using the Fast Fourier transformation algorithm, were derived from the raw signal.

### 2.6. Anthropometry and Body Composition Assessments

The participant’s waist circumference (measured with a plastic tape), BW, and height (determined using a SECA model 799; Hamburg, Germany) were assessed before the gas exchange measurement. Subsequently, participants’ whole-body fat and lean masses, and fat percentage were obtained by using a Dual Energy X-ray Absorptiometry scanner (Discovery Wi, Hologic Inc., Bedford, MA, USA).

### 2.7. Statistical Analyses

The v.22.0 of the Statistical Package for the Social Sciences software (IBM SPSS Statistics, IBM Corporation, Chicago, IL, USA) was used to perform the statistical analyses. A threshold of *p* ≤ 0.05 was considered statistically significant. Figures were created using GraphPad, v. 8.0.2 software (San Diego, CA, USA). The variables that presented a non-Gaussian distribution (checked by performing a visual inspection of histograms and the Kolmogorov–Smirnov test) were transformed and used in the analyses with natural logarithm (ln). Sex differences were reported for all the health-related parameters using non-paired t-tests. Analysis of covariance (ANCOVA) was conducted to explore sex-adjusted differences (age as a covariate) for the main variables (i.e., gas exchange parameters [CVs for VO_2_, VCO_2_, RER, and REE]). Multiple linear regressions analyses were performed to examine the associations between the above-mentioned CVs and (i) anthropometry and body composition parameters; (ii) cardiorespiratory fitness; (iii) BP and circulating cardiometabolic risk factors; and (iv) HR and vagal-related HRV parameters. Furthermore, we performed the associations between the CVs derived from gas exchange and cardiometabolic risk Z-scores 1 and 2. Age was considered a covariate in the statistical models, and all the analyses were performed in men and women separately. Unless otherwise stated, the results are reported as mean ± standard deviation (SD) for descriptive purposes and as standardized β in linear regressions.

## 3. Results

A total of 34 men (54 ± 5 years old) and 38 women (53 ± 5 years old) participated. Table 1 shows sample characteristics for men and women separately. Men showed higher weight, height, BMI, lean mass, waist circumference, and cardiorespiratory fitness but lower fat mass (in %) values than women (*p* ≤ 0.001; Table 1). Likewise, men presented an adverse cardiometabolic risk profile compared to women, specifically for HDL-C, BP, and cardiometabolic risk Z-scores (*p* ≤ 0.041; Table 1). HR and vagal-related HRV parameters were similar between men and women (*p* > 0.050; Table 1).

**Figure 1:**
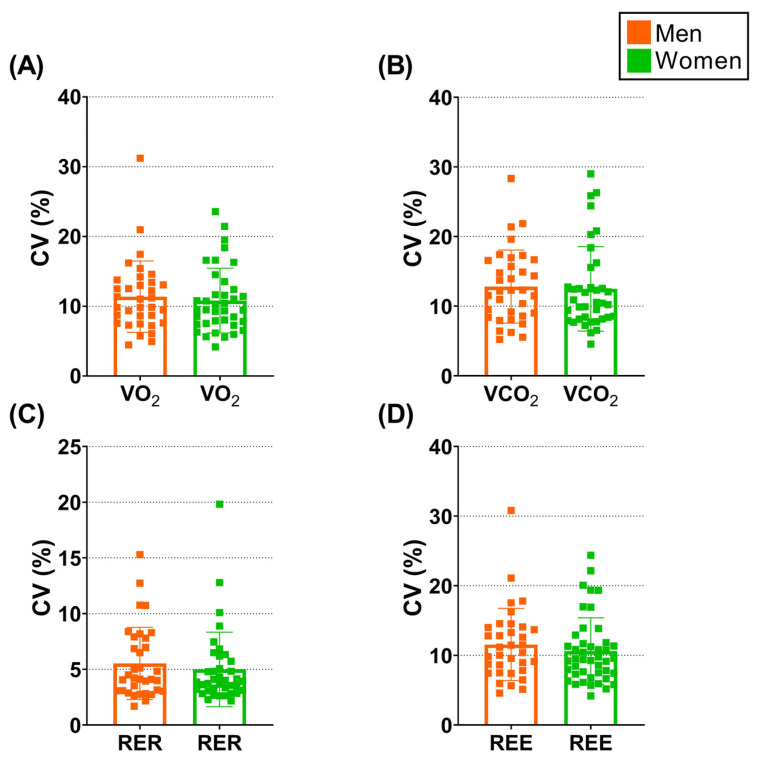
Column dot-plots for coefficient of variation (CV, in %) data separated by men (orange squares) and women (green squares). CVs were calculated for each individual and gas exchange parameter, i.e., for the volumes of oxygen consumption (VO_2_; Panel (**A**)) and carbon dioxide production (VCO_2_; Panel (**B**)), for the respiratory exchange ratio (RER; Panel (**C**)), and for the resting energy expenditure (REE; Panel (**D**)), separately. *n* = 34 men and 38 women. Results are presented as mean and standard deviation and individual data points (depicted as squares).

Figure 1 shows the mean intra-assessment variability and the individual CVs for VO_2_, VCO_2_, RER, and REE for men and women separately. ANCOVA analyses showed no significant differences between sexes (all *p* ≥ 0.549), suggesting that men and women presented similar CVs for VO_2_ (adjusted mean difference of 0.23%; Figure 1A), VCO_2_ (adjusted mean difference of 0.04%; Figure 1B), RER (adjusted mean difference of 0.48%; Figure 1C), and REE (adjusted mean difference of 0.21%; Figure 1D).

Table 2 shows the results derived from multiple linear regression analyses of the intra-assessment resting metabolic rate variability-related parameters with anthropometry, body composition, and CRF. In men, a positive association of the CV for RER with BMI and fat mass (both β = 0.35, both *p* ≤ 0.048, Table 2) was observed, while the CVs for VCO_2_ and REE were negatively associated with whole-body fat mass (both β = −0.36 and −0.36, both *p* ≤ 0.041, Table 2) in women.

Table 3 shows the results derived from multiple linear regression analyses of the intra-assessment resting metabolic rate variability-related parameters with BP and circulating cardiometabolic risk factors. In men, a negative association of the CV for VO_2_ and for RER with total cholesterol and LDL-C (β ranged from −0.37 to −0.46, all *p* ≤ 0.036, Table 3) was observed, while the CV for VCO_2_ was negatively associated with LDL-C (β = −0.39, *p* = 0.025, Table 3). In women, the CV for VCO_2_ and the CV for REE were negatively associated with the cardiometabolic risk score 1 (β = −0.40 and −0.38, both *p* ≤ 0.05, Table 3).

## 4. Discussion

**Figure 2:**
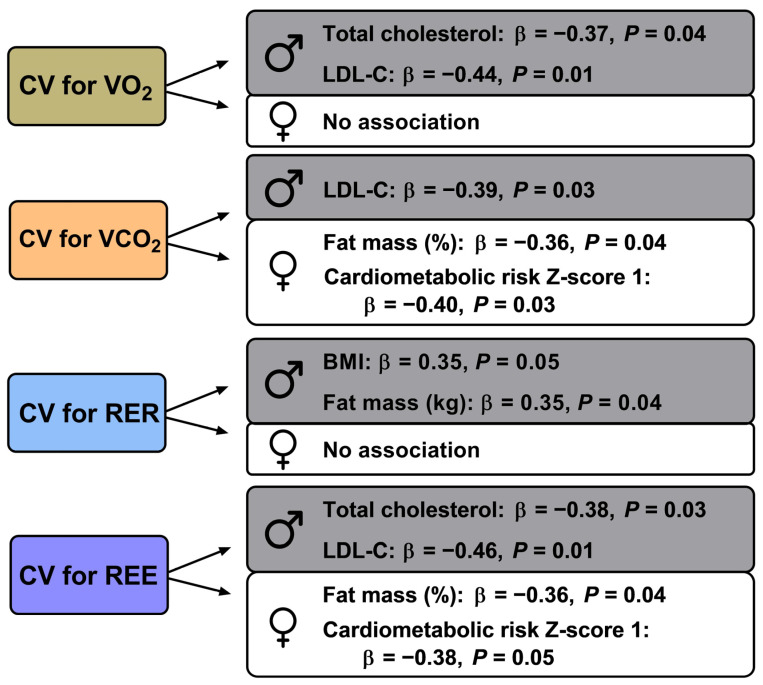
Summary of key findings of the study retrieved by linear regression analyses investigating the relationship between resting metabolic rate variability parameters (approached as CV for VO_2_, VCO_2_, RER, and REE) and classic cardiometabolic risk factors. The figure highlights standardized β and *p* values from multiple linear regression analyses (adjusted for age).

In the present manuscript, we explored the CVs for VO_2_, VCO_2_, RER, and REE as surrogates of intra-assessment resting metabolic rate variability yielded by middle-aged adults, and its association with a range of health-related markers. In summary, we observed a similar intra-assessment resting metabolic rate variability between men and women. Moreover, we showed that the CV for VO_2_, VCO_2_, RER, and REE were associated with health-related markers (BMI, fat mass, total cholesterol, LDL-C, and a cardiometabolic risk Z-score that includes glucose, triglycerides, HDL-C, BP, and waist circumference) in middle-aged adults independently of their sex. These main findings are summarized below, in Figure 2.

It is well known that BW and body composition (e.g., lean and fat-free masses) are the main contributors to REE [32,33]. Recent literature has demonstrated that men exhibit higher variability in REE compared to their women counterparts [12]. These observations remained after accounting for potential confounders such as age, height, fat mass, and fat-free masses [12]. Moreover, these results were confirmed by a recent study conducted in our laboratory [9], in which we noted that young men presented larger CVs for VO_2_, VCO_2_, and REE compared to young women. Therefore, considering this previous evidence, it seems plausible that middle-aged men would exhibit a larger intra-assessment resting metabolic rate variability compared to women with similar biological characteristics. However, the current data show no CV differences between sexes (Figure 1). Interestingly, the magnitude of the variability exhibited by our middle-aged adult sample was, at least apparently, similar to that yielded by young adults in our previous study [9]. On the other hand, in the present study, we observed that middle-aged men were heavier and taller, and presented higher BMI and lean mass values (Table 1) compared to women. We reported a positive association between CVs for RER and BMI and fat mass (in kg) in men, while a negative association between CVs for VCO_2_ and REE with fat mass was found in women. Thus, these results may suggest a relationship between the intra-assessment variability and whole-body fatness. Conversely, no relationship was detected between the intra-assessment resting metabolic rate variability and the cardiorespiratory fitness status in men or women. This absence of relationship could be explained by the biological characteristics of our study sample (i.e., middle-aged adults) since previous studies have pointed out that regular physical activity directly and positively influences cardiometabolic health, tissues, and organs [34].

Our findings pointed out that there was no association between the intra-assessment resting metabolic rate variability and BP, an issue that was consistent in both men and women. This result could be explained, at least partially, by the fact that the participants yielded high-normal (men) and normal (women) BP according to the cut-off points proposed by current guidelines [35]. On the other hand, in men, circulating cardiometabolic risk factors (concretely, LDL-C and total cholesterol) were inversely associated with intra-assessment resting metabolic rate variability. However, no such associations were observed in women, except the CV for REE, which was negatively associated with the cardiometabolic risk Z-score 1. These results seem to have relevant practical and clinical implications since they suggest that the intra-assessment REE variability could be a potential indicator of cardiometabolic health in middle-aged adults. Of note, these findings do not concur with those observed in young adults, as a relationship of intra-assessment variability with circulating cardiometabolic risk factors was retrieved [9]. Nevertheless, the controversial study findings might be due to the participants’ age (young vs. middle-aged adults) and their differences in cardiometabolic health status. Indeed, the cardiometabolic profile of these young adults included in [9] did not improve after 24 weeks of supervised exercise training [36], while a significant improvement was obtained in response to a 12-week supervised exercise intervention in the middle-aged adult sample [37] included in the present study. These discrepancies could be attributed to the contrasting characteristics of middle-aged adults compared to their younger counterparts. Middle-aged adults, typically presenting suboptimal cardiometabolic health, may exhibit a greater potential for improvement and a higher probability of significant cardiometabolic health benefits following physical exercise interventions compared to younger populations. Therefore, the observed variations in the study outcomes might be elucidated by the disparities associated with the natural aging process and lifestyle factors prevalent within this population.

Contrary to our expectations, we did not find any associations of intra-assessment resting metabolic rate variability with HR and vagal-related HRV parameters in our study sample. Moreover, the intra-assessment resting metabolic rate variability—approached as the CV for RER—was positively associated with HR, and negatively associated with RMSSD and pNN50 (both vagal-related HRV parameters) in young men [9]. A meta-analysis of prospective studies [24] has also suggested that those individuals yielding higher HR values presented an increased risk of suffering from a wide range of cardiometabolic complications and all-cause mortality. This is in line with the scientific literature linking vagal-related HRV parameters and cardiometabolic health [25]. Thus, we hypothesized that age and cardiometabolic health status may explain discrepancies between the present and our previous study [9], as we mentioned above. In fact, HR increases with age, while HRV exhibits a significant decrement during the aging process, these arguments could partially explain the abovementioned discrepancies [38,39,40,41,42]. Finally, considering the observed associations of the intra-assessment resting metabolic rate variability with the health indicators included in this study, we suggest its inclusion in future studies as a potential indicator of cardiometabolic health. As we acknowledge here and in our previous study [9], approaching intra-assessment resting metabolic rate variability using the CVs is a feasible procedure that does not require additional measurements, also presenting the advantage that it can be retrospectively computed following specific procedures [9].

## 5. Clinical and Practical Implications 

Indirect calorimetry offers a non-invasive method for assessing gas exchange in both spontaneously breathing patients and those utilizing mechanical ventilation [43,44,45]. Moreover, this technique can determine RER from gas exchange measurements, a calculation that facilitates determining substrate oxidation rates for glucose and lipids [4,46]. In the context of patients with chronic illnesses or intensive care unit patients, REE may constitute most of their energy requirements, while the RER reflects the predominance of substrates being oxidized. This information proves valuable for tailoring nutrition regimens, ensuring alignment between energy intake and needs [43,44,45]. Moreover, for critically ill patients, indirect calorimetry provides the possibility to observe and monitor metabolic alterations across time. Thus, periodic gas exchange measurements are often conducted to monitor dynamic changes and optimize the energy prescription and energy intake of these patients. In this scenario, the variability of gas exchange parameters (i.e., CV for VO_2_, VCO_2_, RER, and REE) can be retrospectively computed as detailed in our manuscript and used to monitor the patient’s health status evolution non-invasively. Indeed, the variability in gas exchange parameters might be used as a complementary parameter for decision-making, as it could reflect whether the recommendations and adjustments made by the clinicians are aligned with the improvements in their health status. It should be noted, however, that this variability itself would not replace any of the classical cardiometabolic indicators, and should be considered as another indicator of cardiometabolic health. In this regard, computing and using the resting metabolic rate variability could be of great importance for these centers (e.g., research centers) in which measurements of blood circulating cardiometabolic parameters, heart rate rhythm, or body composition (using DXA or bioimpedance analyses) are not available, as we showed here that there is a relationship between resting metabolic rate variability and these parameters in middle-aged adults. Nevertheless, future randomized controlled trial studies and longitudinal designs are warranted in healthy and diseased populations to determine the clinical implications of our results and the inclusion of resting metabolic rate variability assessment in the clinical context.

Finally, the present study has certain limitations that should be highlighted. The assessment of gas exchange was performed using the CPX Ultima CardiO2 metabolic cart and a face mask. Thus, we cannot ascertain whether the use of other metabolic carts or gas collection systems could influence the results. The cross-sectional nature of the study prevents us from establishing causality. In this regard, future studies with different designs (e.g., repeated measures, longitudinal) are needed to corroborate or contrast our findings. Our study was conducted in relatively healthy middle-aged adults; thus, future studies performed in other populations (e.g., individuals with obesity or related metabolic complications) using larger sample sizes are warranted. We did not assess the cost-effectiveness of measuring resting metabolic rate variability, thus its impact on patient care and the economy (e.g., hospital economy) remains unknown. Finally, although we used a valid heart rate monitor to record heart rhythm [47], we could not assess HR and vagal-related HRV parameters using the gold-standard instrument (i.e., an electrocardiograph). 

## 6. Conclusions

In conclusion, the present study conducted in middle-aged adults shows no between-sex differences in intra-assessment resting metabolic rate variability. Nevertheless, in men, CVs for VO_2_, VCO_2_, and REE were negatively associated with total cholesterol and LDL-C, while the CV for RER was positively associated with BMI and fat mass. In contrast, in women, the CV for VCO_2_ and the CV for REE were negatively associated with fat mass and the cardiometabolic risk Z-score. Therefore, our results suggest that intra-assessment resting metabolic rate variability could be used as a non-invasive indicator of cardiometabolic health in middle-aged adults.

## Figures and Tables

**Table 1:** Sample characteristics separated by men and women.

	Men (*n* = 34)		Women (*n* = 38)		
	Mean	SD	Mean	SD	*p*
Anthropometry and body composition					
Weight (kg)	87	11	65	9	**<0.001**
Height (cm)	176	6	161	6	**<0.001**
BMI (kg/m^2^)	28	4	25	3	**<0.001**
Fat mass (kg)	31	10	29	7	0.440
Fat mass (%)	34	8	45	7	**<0.001**
Lean mass (kg)	54	6	34	6	**<0.001**
Waist circumference (cm)	103	9	88	10	**<0.001**
Cardiorespiratory fitness					
CRF (mL/min)	2920	378	1790	314	**<0.001**
CRF_BW_ (mL/[kg/BW]/min)	33	5	28	5	**<0.001**
CRF_LM_ (mL/[kg/lean mass]/min)	54	7	53	9	0.652
Blood pressure and circulating cardiometabolic risk factors					
Systolic BP (mm Hg)	134	14	121	15	**0.001**
Diastolic BP (mm Hg)	85	11	78	12	**0.009**
Glucose (mg/dl)	95	14	93	9	0.450
Insulin (UI/mL)	9	7	8	5	0.466
HOMA index	2	2	2	1	0.357
Total cholesterol (mg/dL)	202	36	211	30	0.231
HDL-C (mg/dL)	56	13	62	12	**0.041**
LDL-C (mg/dL)	126	31	127	27	0.958
Triglycerides (mg/dL)	147	85	126	50	0.213
Cardiometabolic risk Z-score 1	0.4	0.9	−0.3	0.5	**<0.001**
Cardiometabolic risk Z-score 2	0.2	0.8	−0.2	0.5	**0.014**
Heart rate and heart rate variability					
HR (bpm)	62	10	64	7	0.353
RMSSD (ms)	29	17	34	21	0.361
SDNN (ms)	30	14	31	14	0.669
pNN50 (%)	13	16	13	17	0.917
HF (ms^2^)	374	389	548	754	0.230

Results are presented as mean and standard deviation (SD), and *p* values were obtained from unpaired *t*-student analyses. Bold numbers show statistically significant differences (*p* < 0.05).

**Table 2:** Relationship between coefficient of variation (CV) for volumes of oxygen consumption (VO_2_) and carbon dioxide production (VCO_2_), for respiratory exchange ratio (RER), and for resting energy expenditure (REE) and anthropometry and body composition, and cardiorespiratory fitness (CRF) parameters.

	CV for VO_2_				CV for VCO_2_				CV for RER				CV for REE			
	Men		Women		Men		Women		Men		Women		Men		Women	
	β	*p*	β	*p*	β	*p*	β	*p*	β	*p*	β	*p*	β	*p*	β	*p*
Anthropometry and body composition																
Weight (kg)	0.14	0.41	−0.04	0.84	0.18	0.30	−0.08	0.65	0.28	0.10	−0.13	0.45	0.14	0.39	−0.06	0.76
Height (cm)	0.10	0.57	0.22	0.21	0.03	0.87	0.21	0.21	−0.16	0.35	0.08	0.63	0.06	0.71	0.21	0.22
BMI (kg/m^2^)	0.11	0.56	−0.17	0.36	0.18	0.33	−0.21	0.25	**0.35**	**0.05**	−0.18	0.30	0.13	0.48	−0.19	0.31
Fat mass (kg)	0.01	0.95	−0.24	0.19	0.11	0.52	−0.29	0.11	**0.35**	**0.04**	−0.12	0.49	0.02	0.92	−0.27	0.14
Fat mass (%)	−0.07	0.70	−0.33	0.06	0.04	0.82	**−0.36**	**0.04**	0.34	0.06	−0.09	0.62	−0.06	0.73	**−0.36**	**0.04**
Lean mass (kg)	0.23	0.19	0.23	0.20	0.14	0.44	0.22	0.21	−0.06	0.75	−0.06	0.73	0.23	0.19	0.23	0.18
Waist Circumference (cm)	0.01	0.98	−0.01	0.96	0.10	0.57	−0.10	0.60	0.31	0.07	−0.16	0.35	0.02	0.90	−0.04	0.83
Cardiorespiratory fitness																
CRF (mL/min)	0.16	0.38	0.12	0.55	0.13	0.46	0.14	0.47	0.13	0.49	0.13	0.46	0.17	0.35	0.13	0.51
CRF_BW_ (mL/[kg/BW]/min)	0.15	0.41	0.22	0.27	0.09	0.62	0.25	0.19	−0.05	0.77	0.26	0.14	0.16	0.40	0.23	0.23
CRF_LM_ (mL/[kg/lean mass]/min)	0.01	0.99	−0.02	0.94	0.05	0.79	−0.01	0.98	0.22	0.24	0.24	0.19	0.01	0.98	−0.02	0.92

Results are presented as standardized β and *p* values from multiple linear regression analyses (adjusted for age). Bold numbers show statistically significant associations (*p* < 0.05).

**Table 3:** Relationship between coefficient of variation (CV) for volumes of oxygen consumption (VO_2_) and carbon dioxide production (VCO_2_), for respiratory exchange ratio (RER), and for resting energy expenditure (REE) and blood circulating cardiometabolic risk factors and pressure, heart rate (HR) and vagal-related heart rate variability (HRV) parameters.

	CV for VO_2_				CV for VCO_2_				CV for RER				CV for REE			
	Men		Women		Men		Women		Men		Women		Men		Women	
	β	*p*	β	*p*	β	*p*	β	*p*	β	*p*	β	*p*	β	*p*	β	*p*
Blood pressure and circulating cardiometabolic risk factors																
Systolic BP (mm Hg)	−0.15	0.44	−0.14	0.46	−0.16	0.39	−0.18	0.35	−0.18	0.35	−0.09	0.63	−0.18	0.35	−0.16	0.42
Diastolic BP (mm Hg)	−0.21	0.28	−0.22	0.26	−0.14	0.48	−0.23	0.22	−0.05	0.80	−0.06	0.72	−0.23	0.24	−0.23	0.25
Glucose (mg/dL)	−0.15	0.42	−0.31	0.09	−0.08	0.68	−0.27	0.13	−0.02	0.92	−0.05	0.79	−0.12	0.50	−0.30	0.10
Insulin (UI/mL)	−0.11	0.55	−0.23	0.20	−0.07	0.69	−0.16	0.37	0.07	0.73	0.13	0.48	−0.11	0.54	−0.23	0.21
HOMA index	−0.13	0.49	−0.28	0.13	−0.08	0.66	−0.2	0.27	0.06	0.77	0.11	0.52	−0.13	0.5	−0.27	0.14
Total cholesterol (mg/dL)	**−0.37**	**0.04**	−0.16	0.39	−0.31	0.08	−0.25	0.15	−0.15	0.40	−0.15	0.40	**−0.38**	**0.03**	−0.20	0.27
HDL-C (mg/dL)	0.10	0.60	0.20	0.27	0.13	0.48	0.25	0.15	0.20	0.28	0.07	0.71	0.13	0.48	0.23	0.21
LDL-C (mg/dL)	**−0.44**	**0.01**	0.05	0.80	**−0.39**	**0.03**	−0.13	0.48	−0.22	0.22	−0.21	0.23	**−0.46**	**0.01**	−0.02	0.91
Triglycerides (mg/dL)	−0.03	0.87	−0.26	0.16	−0.11	0.57	−0.29	0.10	−0.21	0.26	−0.19	0.28	−0.07	0.71	−0.29	0.11
Cardiometabolic risk Z-score 1	−0.26	0.19	−0.37	0.06	−0.21	0.30	**−0.40**	**0.03**	−0.14	0.48	−0.19	0.29	−0.26	0.19	**−0.38**	**0.05**
Cardiometabolic risk Z-score 2	−0.36	0.07	−0.30	0.13	−0.29	0.13	−0.35	0.07	−0.15	0.45	−0.21	0.25	−0.36	0.06	−0.31	0.11
HR and HRV parameters																
HR (bpm)	−0.33	0.06	−0.16	0.38	−0.20	0.25	−0.14	0.44	0.09	0.62	0.02	0.91	−0.30	0.09	−0.15	0.41
RMSSD (ms)	0.08	0.64	0.03	0.85	−0.06	0.74	0.01	0.99	−0.18	0.30	0.13	0.46	0.05	0.78	0.01	0.08
SDNN (ms)	0.29	0.08	0.15	0.39	0.15	0.37	0.10	0.57	−0.02	0.92	0.14	0.40	0.26	0.12	0.12	0.50
pNN50 (%)	0.16	0.35	0.04	0.8	0.02	0.92	0.03	0.87	−0.16	0.37	0.13	0.41	0.13	0.47	0.02	0.91
HF (ms^2^)	−0.01	0.96	0.09	0.59	−0.13	0.44	0.06	0.73	−0.17	0.31	0.18	0.28	−0.04	0.81	0.07	0.68

Results are presented as standardized β and *p* values from multiple linear regression analyses (adjusted for age). Bold numbers show statistically significant associations (*p* < 0.05).

## Data Availability

All data are available from the corresponding author upon reasonable request.

## Supplementary Materials

The following supporting information can be downloaded at: https://www.mdpi.com/article/10.3390/jcm12237321/s1, Figure S1: Study participant flow-chart.
