# Supplementary figures and images for: Intra-Assessment Resting Metabolic Rate Variability Is Associated with Cardiometabolic Risk Factors in Middle-Aged Adults
Source: J Clin Med. 2023 Nov 26;12(23):7321. doi: 10.3390/jcm12237321 (PMC10707389; doi:10.3390/jcm12237321)

### Enrollment and analysis flowchart

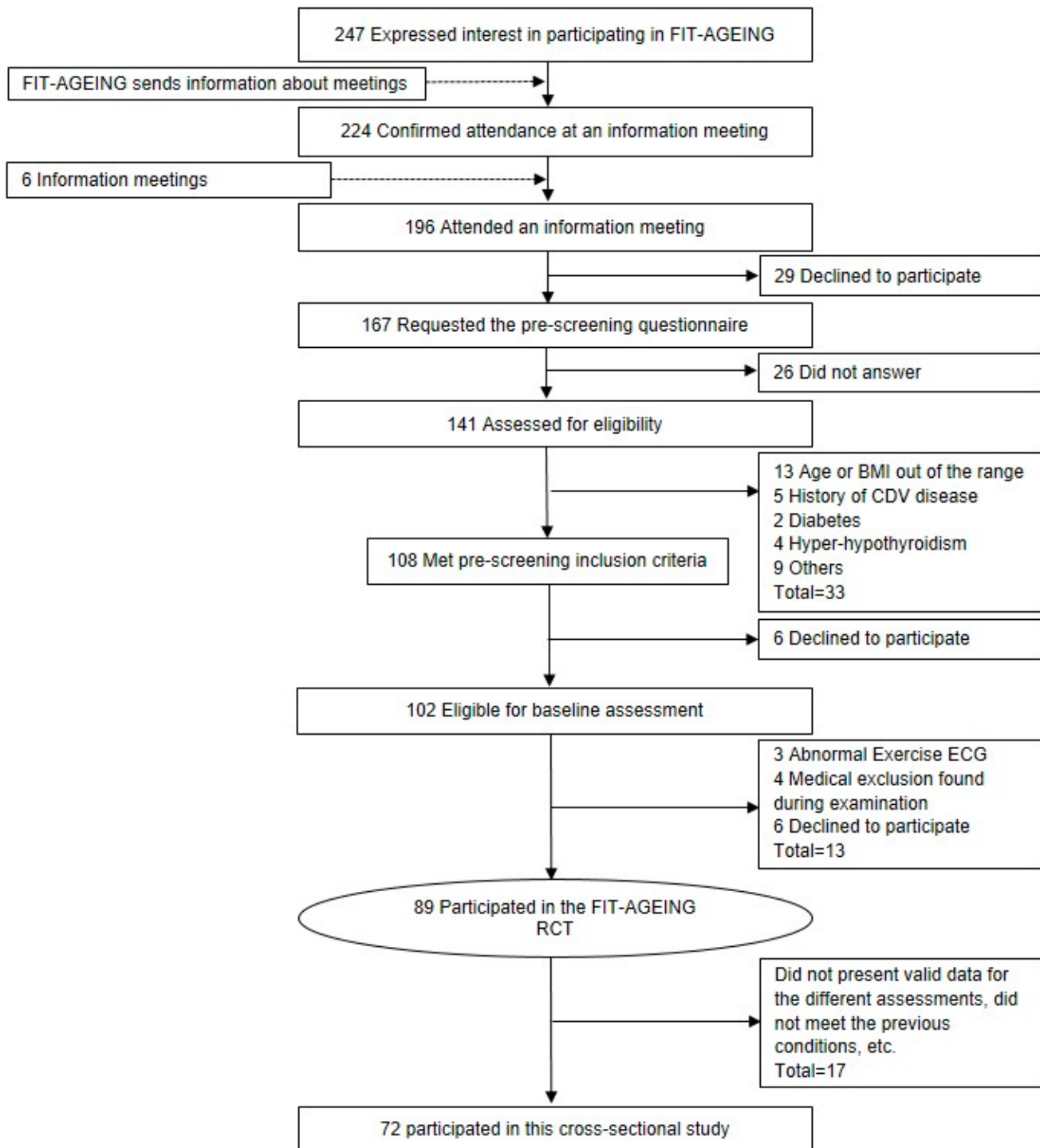

**Figure S1.** Study participant flow-chart.

Supplement: Supplementary file 1 [file jcm-12-07321-s001.zip › jcm-2628038-supplementary.pdf]
